# Prolonged Serum Alanine Aminotransferase Elevation Associated with Isotretinoin Administration

**DOI:** 10.1155/2019/9270827

**Published:** 2019-07-17

**Authors:** Roya S. Nazarian, Elizabeth Zheng, Caroline Halverstam, Steven R. Cohen, Allan W. Wolkoff

**Affiliations:** ^1^Icahn School of Medicine at Mount Sinai, One Gustave L Levy Place New York, NY 10029, USA; ^2^Division of Digestive and Liver Disease, Columbia University, 622 W 168th St, New York, NY 10032, USA; ^3^Division of Dermatology, Albert Einstein College of Medicine and Montefiore Medical Center, 3411 Wayne Avenue, Bronx, NY 10467, USA; ^4^Marion Bessin Liver Research Center, Division of Hepatology, Albert Einstein College of Medicine and Montefiore Medical Center, 1300 Morris Park Avenue, Bronx, NY 10461, USA

## Abstract

Isotretinoin is a highly effective oral retinoid derivative for severe forms of acne. Despite its high margin of safety, isotretinoin carries a risk of teratogenicity and mild to massive elevations of serum cholesterol and triglyceride levels, as well as infrequent transaminitis. Liver dysfunction induced by isotretinoin is rare but it poses a management dilemma. We describe a 16-year-old male in whom alanine aminotransferase (ALT) rose from a baseline of 13 to 288 U/L after 20 weeks of treatment with 1.0-1.4 mg/kg of oral isotretinoin daily. Though the patient remained asymptomatic, ALT levels did not return to normal limits for approximately 8 months after discontinuation of therapy, an observation that has not been documented in the literature. When oral isotretinoin was readministered for intractable facial acne 3 years later, liver enzymes remained normal throughout the course of therapy. Although the pathogenesis and prognosis of retinoid-induced hepatotoxicity are unknown, this case illustrates that isotretinoin may be safely readministered after normalization of liver function tests.

## 1. Introduction

Isotretinoin is a highly effective oral retinoid therapy for severe and recalcitrant forms of nodular and cystic acne [1–3]. Although isotretinoin has a high margin of safety, adverse effects include teratogenicity and elevated serum cholesterol, triglyceride, and transaminase levels [1, 4, 5]. Rare hepatic dysfunction during long-term isotretinoin therapy has not caused liver damage on serial biopsies [5, 6]. Nonetheless, transaminase elevations have been reported as high as 5-fold above baseline normal values [3–5, 7]. We report a 16-year-old male who experienced a rise in serum alanine aminotransferase (ALT) from 13 to 288 U/L during isotretinoin therapy that did not return to baseline for approximately 8 months after cessation of the drug, a phenomenon not previously described in the literature. When intractable cystic acne developed in the same patient three years later, a second 5-month course of isotretinoin was accompanied by normal ALT levels and other parameters of liver function.

## 2. Case Presentation

**Figure 1:**
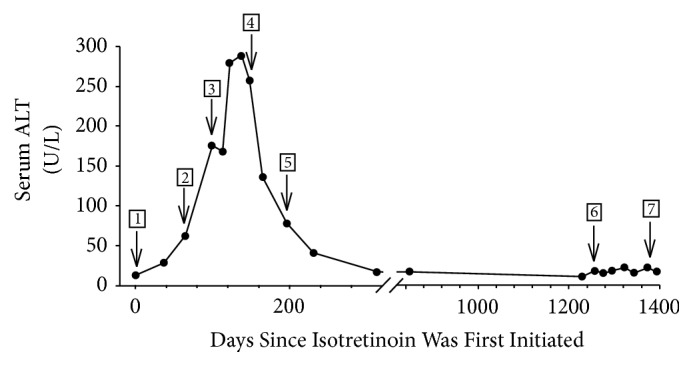
Serum alanine aminotransferase levels (ALT) over time. The numbered arrows represent 1. initiation of oral isotretinoin at 30 mg twice a day; 2. increase in the dose to 40 mg twice a day; 3. discontinuation of isotretinoin; 4. initiation of oral cholestyramine powder 4 g a day with breakfast; 5. discontinuation of cholestyramine; 6. reinstitution of oral isotretinoin at 30 mg twice a day; 7. discontinuation of isotretinoin.

An otherwise healthy 16-year-old boy presented with painful and disfiguring facial acne of several years duration unresponsive to a wide range of topical and oral antibiotic regimens. Physical examination showed extensive, red nodular, and fluctuant cysts with scarring on the face. Baseline blood tests were all within normal limits prior to starting isotretinoin, 30 mg twice a day (1.0 mg/kg) (Figure 1, arrow 1). After two months of therapy, refractory acne prompted an increase of isotretinoin to 40 mg twice a day (1.4 mg/kg). The significance of an elevated ALT to 62 U/L [nl: <30 U/L] was uncertain (Figure 1, arrow 2). By the third month of isotretinoin therapy, there was a dramatic improvement of facial acne; however, ALT levels rose to 175 U/L. Isotretinoin was discontinued (Figure 1, arrow 3). Approximately 4 weeks after stopping isotretinoin, ALT levels reached a peak of 288 U/L, which led to a consultation with Hepatology. A review of systems and physical examination were normal. Laboratory studies showed normal bilirubin (0.5 [nl: 0.0-0.4 mg/dl]), alkaline phosphatase (153 U/L [nl: < 171 U/L]), gamma glutamyl transpeptidase (38 U/L [nl: < 54 U/L]), ferritin (104 ng/ml [nl: < 270 ng/ml]), INR (1.2 [nl: <1.1]), and platelet count (324,000/*µ*l [nl: 150,000-400,000]). Lipid studies showed normal levels of cholesterol (120 mg/dl [nl: <200 mg/dl]) and triglycerides (54 mg/dl [nl: <150 mg/dl]). Screening for hepatitis A, B, and C, as well as antinuclear antibodies, was negative. Considering enterohepatic recycling of potential hepatotoxic isotretinoin metabolites, the patient was started on cholestyramine powder, 4g daily with breakfast (Figure 1, arrow 4). This was discontinued after 7 weeks as ALT levels were returning towards normal (Figure 1, arrow 5). The ALT levels completely returned to normal values one month later and returned to baseline 2 months thereafter (Figure 1).

Because of worsening cystic acne unresponsive to topical retinoids, topical antibiotics, and oral doxycycline, another course of isotretinoin was started approximately 3 years later (Figure 1, arrow 6). During the ensuing 5 months of treatment, ALT and other liver function tests remained normal (Figure 1, arrow 7). All inflammatory and cystic acne resolved by the conclusion of treatment.

## 3. Discussion

Oral isotretinoin is a highly effective therapy for severe cystic acne [1, 3]. Since its approval by the US Food and Drug Administration in 1982, an estimated 12 million individuals have been treated with this drug [2]. Its efficacy derives from serving as a ligand for the retinoic acid receptor (RAR), a nuclear receptor that forms a heterodimer with the retinoid X receptor (RXR), activating the transcription of target genes [7]. The resulting decrease in sebocyte proliferation suppresses acne by reducing sebaceous gland size and sebum production [1, 8].

Although isotretinoin therapy has a high margin of safety, side effects and complications are well characterized, with the most significant being teratogenicity. Other common side effects are dry skin, eyes, lips, and mucous membranes [1, 2]. Though altered LDL, HDL, and triglyceride levels are also common, the exact mechanism is unknown, and laboratory abnormalities spontaneously resolve after cessation of isotretinoin [2, 6]. Less frequent gastrointestinal side effects include nausea and the potential exacerbation of inflammatory bowel disease, though the latter remains controversial [2, 9, 10].

Hepatotoxicity in cases of hypervitaminosis A has been well documented [6]. By contrast, hepatotoxicity associated with isotretinoin is uncommon [3, 4]. A recent study of high-dose isotretinoin therapy in 80 patients with acne vulgaris revealed minimal, asymptomatic transaminase elevations in 23% of the cohort [3]. While mild liver enzyme elevations during treatment with isotretinoin have been extensively reported, there is a comparable return to baseline (normal) after discontinuation of treatment. Our case is the first to describe persistent ALT elevations after discontinuation of isotretinoin therapy (Figure 1, arrow 3).

The mechanism of isotretinoin-induced hepatotoxicity is obscure, but postulated triggers include the parent compound itself or a toxic metabolite. Isotretinoin is a hydrophobic molecule that is highly bound (>99%) to plasma proteins [11, 12] and metabolized by cytochrome P450 enzymes in the liver [13]. Its serum half-life is approximately 14 hours [14, 15], and the half-life of its major metabolite, 4-oxo-isotretinoin, is closer to 28 hours [15]. Additional metabolites were suggested by one study that followed the administration of an oral dose of ^14^C-isotretinoin in which the serum half-life of labeled ^14^C averaged 90 hours, indicating the presence of other isotretinoin metabolites [15]. By contrast, when ^14^C-isotretinoin was administered to two patients with t-tube drainage of bile, there was a trend towards shortened half-lives, suggesting enterohepatic circulation of isotretinoin metabolites [15]. Our case is unique as the sequestering agent, cholestyramine, was administered to reduce isotretinoin toxicity (Figure 1, arrow 4), associated with the potential accumulation of toxic metabolites recycling in the enterohepatic system. Serum ALT levels subsequently dropped by almost 50% within 2 weeks of starting cholestyramine (Figure 1). The association between normalized liver function tests (LFTs) and cholestyramine therapy remains uncertain; however, ALT levels no longer decreased after discontinuation of cholestyramine 2 months later (Figure 1, arrow 5).

Interpretation of lab abnormalities in the setting of isotretinoin therapy is challenging. In this case, elevated LFT levels may have been due to the high daily dose of isotretinoin. However, studies have shown that liver abnormalities associated with high-dose isotretinoin therapy are rare, occurring in 1.3% of patients [3, 13]. Modest serum ALT elevations associated with isotretinoin therapy are usually self-limited and generally do not require dose modification or discontinuation of therapy [3]. Nonetheless, discontinuation of isotretinoin is recommended when ALT elevations are 3-fold or more above the upper limit of normal, as in our patient [3]. Readministration of isotretinoin following asymptomatic hepatic function lab abnormalities poses a dilemma because there is no evidence-based standard of care. This case demonstrates that isotretinoin may be safely readministered in the context of intractable and disfiguring acne years after isotretinoin-induced lab abnormalities. Following normalization of biochemical parameters, etretinate may be another treatment option since cross-toxicity is not apparent between the various retinoid derivatives [16]. Because of prolonged fat retention, etretinate therapy in women of childbearing potential is limited.

In summary, this case is the first to describe prolonged liver function test abnormalities following use and discontinuation of isotretinoin for acne. Isotretinoin should be withdrawn if ALT elevations are greater than 3 times the normal value. Cholestyramine may expedite normalization of lab values. Finally, this case suggests that readministration of isotretinoin, with close monitoring, after normalization of liver function tests may be a safe option for treatment resistant acne.

## References

[1] Degitz K., Ochsendorf F. (2008). Pharmacotherapy of acne. *Expert Opinion on Pharmacotherapy*.

[2] Merritt B., Burkhart C. N., Morrell D. S. (2009). Use of isotretinoin for acne vulgaris. *Pediatric Annals*.

[3] Cyrulnik A. A., Viola K. V., Gewirtzman A. J., Cohen S. R. (2012). High-dose isotretinoin in acne vulgaris: improved treatment outcomes and quality of life. *International Journal of Dermatology*.

[4] Zane L. T., Leyden W. A., Marqueling A. L., Manos M. M. (2006). A population-based analysis of laboratory abnormalities during isotretinoin therapy for acne vulgaris. *JAMA Dermatology*.

[5] Kizilyel O., Metin M. S., Elmas Ö. F., Çayir Y., Aktas A. (2014). Effects of oral isotretinoin on lipids and liver enzymes in acne patients. *Cutis; Cutaneous Medicine for the Practitioner*.

[6] Roenigk H. H. (1989). Liver toxicity of retinoid theraphy. *Pharmacology & Therapeutics*.

[7] Fallon M. B., Boyer J. L. (1990). Hepatic toxicity of Vitamin A and synthetic retinoids. *Journal of Gastroenterology and Hepatology*.

[8] Zouboulis C. C. (2006). Isotretinoin revisited: pluripotent effects on human sebaceous gland cells. *Journal of Investigative Dermatology*.

[9] Etminan M., Bird S. T., Delaney J. A., Bressler B., Brophy J. M. (2013). Isotretinoin and risk for inflammatory bowel disease: a nested case-control study and meta-analysis of published and unpublished data. *JAMA Dermatology*.

[10] Racine A., Cuerq A., Bijon A. (2014). Isotretinoin and risk of inflammatory bowel disease: a french nationwide study. *American Journal of Gastroenterology*.

[11] Geubel A., De Galocsy C., Alves N., Rahier J., Dive C. (1991). Liver damage caused by therapeutic vitamin A administration: Estimate of dose-related toxicity in 41 cases. *Gastroenterology*.

[12] Thatcher J. E., Isoherranen N. (2009). The role of CYP26 enzymes in retinoic acid clearance. *Expert Opinion on Drug Metabolism & Toxicology*.

[13] Blasiak R. C., Stamey C. R., Burkhart C. N., Lugo-Somolinos A., Morrell D. S. (2013). High-dose isotretinoin treatment and the rate of retrial, relapse, and adverse effects in patients with acne vulgaris. *JAMA Dermatology*.

[14] Colburn W. A., Gibson D. M. (1985). Isotretinoin kinetics after 80 to 320 mg oral doses. *Clinical Pharmacology & Therapeutics*.

[15] Colburn W. A., Vane F. M., Bugge C. J., Carter D. E., Bressler R., Ehmann C. W. (1985). Pharmacokinetics of 14C-isotretinoin in healthy volunteers and volunteers with biliary T-tube drainage. *Drug Metabolism and Disposition: The Biological Fate of Chemicals*.

[16] Marhold I., Duschet P., Schwarz T., Gschnait F. (1991). Successful use of isotretinoin in type Zumbusch generalized pustular psoriasis following recovered etretinate-induced hepatitis. *Der Hautarzt; Zeitschrift fur Dermatologie, Venerologie, und verwandte Gebiete*.

